# Bilaterally Asymmetric Helical Myofibrils in Ascidian Tadpole Larvae

**DOI:** 10.3389/fcell.2021.800455

**Published:** 2021-12-07

**Authors:** Koichi Matsuo, Ryota Tamura, Kohji Hotta, Mayu Okada, Akihisa Takeuchi, Yanlin Wu, Koh Hashimoto, Hidekazu Takano, Atsushi Momose, Atsuo Nishino

**Affiliations:** ^1^ Laboratory of Cell and Tissue Biology, Keio University School of Medicine, Tokyo, Japan; ^2^ Department of Neurosurgery, Keio University School of Medicine, Tokyo, Japan; ^3^ Department of Biosciences and Informatics, Faculty of Science and Technology, Keio University, Yokohama, Japan; ^4^ Japan Synchrotron Radiation Research Institute (JASRI), Sayo, Japan; ^5^ Institute of Multidisciplinary Research for Advanced Materials, Tohoku University, Sendai, Japan; ^6^ Department of Biology, Faculty of Agriculture and Life Science, Hirosaki University, Hirosaki, Japan

**Keywords:** bilateral symmetry, left-right asymmetry, *Ciona robusta*, muscle cell, myofibrils, synchrotron radiation, left-handed helix, cellular homochirality

## Abstract

The locomotor system is highly bilateral at the macroscopic level. Homochirality of biological molecules is fully compatible with the bilateral body. However, whether and how single-handed cells contribute to the bilateral locomotor system is obscure. Here, exploiting the small number of cells in the swimming tadpole larva of the ascidian *Ciona*, we analyzed morphology of the tail at cellular and subcellular scales. Quantitative phase-contrast X-ray tomographic microscopy revealed a high-density midline structure ventral to the notochord in the tail. Muscle cell nuclei on each side of the notochord were roughly bilaterally aligned. However, fluorescence microscopy detected left-right asymmetry of myofibril inclination relative to the longitudinal axis of the tail. Zernike phase-contrast X-ray tomographic microscopy revealed the presence of left-handed helices of myofibrils in muscle cells on both sides. Therefore, the locomotor system of ascidian larvae harbors symmetry-breaking left-handed helical cells, while maintaining bilaterally symmetrical cell alignment. These results suggest that bilateral animals can override cellular homochirality to generate the bilateral locomotor systems at the supracellular scale.

## Introduction

Morphological bilateral symmetry is crucial for movement and locomotion. A bilaterally symmetric locomotor system likely increases fitness for life: insects and birds fly by swinging bilateral wings, and most fish swim by flexing bilateral and midline fins. The morphology of vertebrate musculoskeletal and nervous systems exhibits highly bilateral symmetry, and lateralization of movement develops to allow coordinated unilateral or alternate movement in mammals including humans (Welniarz et al., 2015). In contrast to the wealth of information on left-right asymmetry of internal organs initiated by breaking of bilateral symmetry (Levin and Mercola, 1998; Yoshiba et al., 2012), little is known regarding developmental mechanisms that generate external or musculoskeletal bilateral symmetry or asymmetry (Allard and Tabin, 2009; Grimes, 2019).

The ascidian tunicate *Ciona robusta* (*Ciona intestinalis* type A) is the small animal model organism closest to vertebrates (Procaccini et al., 2011; Razy-Krajka and Stolfi, 2019). The swimming tadpole larva of ascidians is composed of a trunk (also referred to as the “head”) and tail. A century ago, analyzing locomotion of the swimming tadpole larva of the ascidians *Amaroucium* and *Botryllus*, Grave reported clockwise rotational swimming of the larva around its long axis in circular or curved paths and recognized lateral asymmetry of the body and the oblique orientation of the muscle fibrillae (Grave, 1920). *Ciona* larvae also display swimming paths characterized as spiral, curved and random (Sakurai et al., 2004) or erratic circular (Salas et al., 2018). Asymmetries in body shape are, however, thought to be too subtle in ascidian larvae (*Distaplia occidentalis*) to explain body rotation during helical swimming (McHenry, 2001).

Bilateral morphology of the ascidian tail at the cellular scale has been described (Satoh, 2003; Nishino et al., 2011). In the midsagittal plane, the tail exhibits a dorsal neural tube, an axial notochord and a ventral endodermal strand (Corbo et al., 2001) in addition to dorsal and ventral fins. The *Ciona* tail contains ∼70 dorsal nerve cord cells, 36 muscle cells flanking 40 notochord cells, ∼20 endodermal strand cells, and ∼380 epidermal cells (Pasini et al., 2006; Nakamura et al., 2012; Kourakis and Smith, 2015). This small number of cells and simple cell lineage allow investigation of bilateral symmetry/asymmetry of the locomotor system at supracellular, cellular, and subcellular scales. Cell-lineage and three-dimensional cell alignment analyses of ascidians have been published in databases, such as ANISEED (http://www.aniseed.cnrs.fr/) and FABA (for Four-dimensional Ascidian Body Atlas, https://www.bpni.bio.keio.ac.jp/chordate/faba/1.4/top.html). Muscle cells undergo their final cell division at the neurula stage, and no proliferation occurs during the period of tail extension, resulting in formation of 18 mononucleated muscle cells on each side of the notochord (Passamaneck et al., 2007). The 8-cell stage *Ciona* embryo contains bilaterally symmetric left-right pairs of A4.1 (vegetal-anterior), B4.1 (vegetal-posterior), a4.2 (animal-anterior), and b4.2 (animal-posterior) blastomeres. Of the 18 muscle cells on each side, 14 primary muscle cells from the anterior end are produced from the ipsilateral B4.1 blastomere (Razy-Krajka and Stolfi, 2019). Thus, the first cleavage separates left and right primary muscle cells in the tail (Hotta et al., 2007). It is worth noting that four muscle cells near the tail tip (called secondary muscle cells) are descendants of A4.1 and b4.2 blastomeres, and the contribution of contralateral b4.2-descendants can frequently be detected (Nishida and Satoh, 1983).

Connectome analyses of the ascidian tadpole brain indicate that the nervous system, which controls muscle tension, exhibits sidedness (Ryan et al., 2016). Brain-controlled muscle contraction results in swimming behavior, including symmetric and asymmetric tail flexions that occur at 10–40 Hz (Nishino et al., 2010; Nishino et al., 2011). A gravity-sensing otolith is located on the midsagittal plane, and a single light sensor ocellus is located on the right trunk (Yoshida and Saiga, 2011). In *Ciona* tailbud stage embryos, heart precursor cells cluster bilaterally, fuse symmetrically at the ventral midline, and then undergo a lateral shift to the right side in early larval stages (Palmquist and Davidson, 2017).

In this study, we asked whether the *Ciona* muscular system is bilaterally symmetric at cellular and subcellular scales. We observed left-handed helical myofibrils in both left and right muscle cells, suggesting that molecular mechanisms in *Ciona* generate a bilaterally symmetric locomotor system at the supracellular scale, overriding cellular homochirality.

## Materials and Methods

### Ascidian Larvae

Ascidian adults *Ciona robusta* (*C. intestinalis* Type A) were provided by the National Bio-Resource Project (NBRP), Japan. Eggs were typically dechorionated in artificial sea water (ASW, Red Sea Salt) containing 0.05% actinase E (Kaken Pharmaceutical Co., Ltd., Japan) and 1% sodium thioglycolate (pH 10, adjusted with 2 M NaOH). Dechorionated eggs were washed in ASW, and sperm from other individuals was added in gelatin-coated 6 cm dishes. When indicated, chorionated eggs were used for *in vitro* fertilization. Fertilized eggs were incubated in gelatin-coated 10 cm dishes at 18°C for 16 to 24 hpf to establish tadpole larvae (Hotta et al., 2007; Hotta et al., 2020). For X-ray imaging, tadpole larvae were immersed 2 h at room temperature (RT) in prefix solution [2% glutaraldehyde, 2% paraformaldehyde (PFA) in 30 mM HEPES buffer, pH7.4, containing 100 mM NaCl and 2 mM CaCl_2_] and stored at 4°C in 10-fold diluted prefix solution in 30 mM HEPES buffer. After rinsing twice in HEPES buffer, larvae were either stained for 1 h at RT in 1% OsO_4_ (TAAB) in 30 mM HEPES or left unstained. Samples were dehydrated in increasing concentrations of ethanol and dried using a critical point drying machine (EM CPD300, Leica, Germany) or on a t-butyl alcohol freeze dryer (VFD-21S, Vacuum Device, Japan). For fluorescence imaging, larvae were fixed overnight in 4% PFA and stored in PBS at 4°C until stained with Alexa Fluor 488 phalloidin (1:50 dilution, A12379, Thermo Fisher Scientific) and DAPI (1:500 dilution, SIGMA-Aldrich) in PBS containing 0.1% Triton X-100 prior to imaging.

### Quantitative Phase-Contrast X-Ray Tomographic Microscopy

Dried samples were sucked into borosilicate glass-capillaries (GD-1.5; 1.5 mm OD, 1.0 mm I.D.; Narishige Scientific Laboratory, Japan), which had been pulled using a horizontal micro-electrode puller (PN-3, Narishige). Phase-contrast X-ray microtomography with Talbot interferometry (hereafter referred to as quantitative X-ray tomographic microscopy) (Takano et al., 2020) was performed at beamline BL37 of a SPring-8 synchrotron radiation facility (Super Photon ring-8 GeV, Hyogo, Japan). A monochromatised X-ray beam at a 9 keV photon energy was used for tomography (2.5 s/projection, 180 projections/180°). A differential phase image at every projection was obtained through a fringe scanning measurement (Takeda et al., 2008) with five steps (0.5 s exposure per step) using the stepping-error correction algorithm (Hashimoto et al., 2020). The effective pixel size at the object plane was 187 nm/pixel. A tomographic image from differential phase images was reconstructed using a filtered backprojection method with a Hilbert filter. Since the field of view was smaller than a single larva, three scans were performed by shifting the larva vertically to cover the entire body, and the resultant image is presented after stitching.

### Light-Sheet Fluorescence Microscopy

Larvae were clarified in 50% glycerol/PBS and mounted in 1% liquid agarose into a 10 μL-glass capillary (Orange marker, inner 0.65 mm, outer 1.2 mm, 701902, BRAND) using a stainless-steel plunger (701930, BRAND). After solidification, the agarose cylinder containing the sample was extruded from the capillary for imaging in Lightsheet Z1 (Carl Zeiss, Germany). The objective lens used was Clr Plan-Apochromat 20x/1.0 with correction ring nd = 1.380. Scaling was 0.32 μm/pixel × 0.32 μm/pixel, and the image size was 610 μm × 610 μm. Z-stack acquisition was performed.

### Confocal Laser Scanning Microscopy

A confocal laser scanning microscope LSM-980 with Airyscan 2 (Carl Zeiss) was used with objective lenses LD LCI Plan-Apochromat 25x/0.8 Imm Korr DIC M27 (scaling was 0.083 μm/pixel × 0.083 μm/pixel, and image size was 644 μm × 338 μm) and C Plan-Apochromat 63x/1.40 (scaling was 0.035 μm/pixel × 0.035 μm/pixel × 0.035 μm/pixel. Image size was 44.5 μm × 44.5 μm).

### Transmission Electron Microscopy

TEM images were acquired using a transmission electron microscope (JEM-1001, JEOL, Japan) at 80 kV, as previously reported (Terakubo et al., 2010). Briefly, larvae were fixed in 2.5% glutaraldehyde in 0.1 M sodium cacodylate buffer (pH 7.4) with 0.35 M sucrose for 1 h at RT, washed with cacodylate buffer, post-fixed in 2% osmium in cacodylate buffer for 1 h, and then dehydrated twice for 5 min each with 50, 70, 90, 99.5, and 100% ethanol. Ultrathin sections of epoxy resin-embedded larvae were stained with uranyl acetate and lead citrate.

### Zernike Phase-Contrast X-Ray Tomographic Microscopy

Laboratory-based Zernike phase-contrast X-ray tomographic microscopy (hereafter referred to as Zernike X-ray tomographic microscopy) (ZEISS Xradia 800 Ultra, Carl Zeiss X-Ray Microscopy, United States) was used with Cu-Kα X-rays (8.04 keV) for tomographic measurement (120 s/projection, 360 projections/180°). The effective pixel size at the object plane was 64 nm, and the image size was 65 μm × 65 μm. A tomographic image was reconstructed using a filtered backprojection method. Synchrotron-based Zernike X-ray tomographic microscopy (Takeuchi et al., 2018; Takeuchi et al., 2021) was also used at beamline BL20XU at SPring-8 (Suzuki et al., 2004) with a monochromatized X-ray beam of 30 keV energy for tomography (100 ms/projection, 900 projections/180°). The effective pixel size at the object plane was 124 nm (31 nm/pixel, 4 × 4 binning), and the image size was 55 μm × 55 μm. Imaging was optimized by varying energy (20 or 30 keV).

### Software

For image analysis, Imaris (version 8, RRID:SCR_007370), ZEN (ZEISS Efficient Navigation, Carl Zeiss), ImageJ (version 1.53) distributed by Fiji (RRID:SCR_002285) (Schindelin et al., 2012), and TRI/3DBON (FCS64, Ratoc System Engineering, Japan) software were used. For visualization of myofibrils, the plasma membrane of muscle cells was traced with nine-pixel-width lines on serial cross sections of laboratory-based Zernike X-ray tomographic microscopy. Using the mask of plasma membrane, we performed segmentation of myofibrils. To generate a schematic model, FreeCAD (version 0.19, available from http://www.freecadweb.org) was used. Adobe Photoshop (RRID:SCR_014199) was used for pseudo-coloring of TEM images.

## Results

### Tadpole Larvae Exhibit High-Density Structures at the Midplane

**FIGURE 1:**
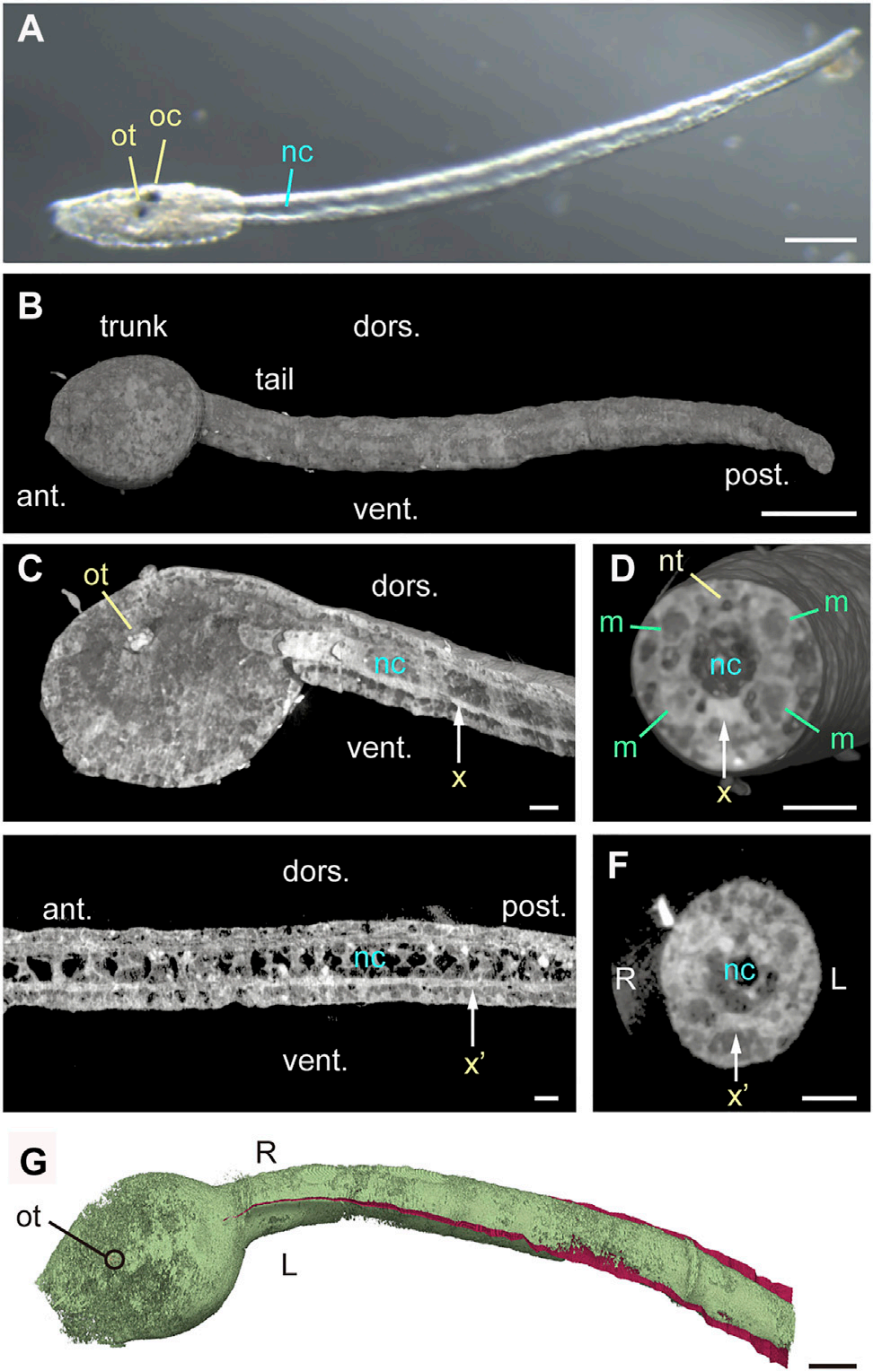
Midplane high-density structures in *Ciona* tadpole larvae. **(A)** Bright field image of 18-hpf larva with elongated tail. Scale bar, 100 μm ot, otolith. oc, ocellus. nc, notochord. **(B)** Quantitative X-ray tomographic microscopy of an osmium-stained 16-hpf larva (left side view). ant., anterior. post., posterior. dors., dorsal. vent., ventral. Scale bar, 50 μm. **(C)** Left view of midsagittal section of 16-hpf larva (cut-view in silico). Vertical arrow (x) indicating high-density ventral structure shows the position of cutting plane in **(D)**. Scale bar, 10 μm. **(D)** Anterior view of a vertical cross-section. nt, neural tube. m, muscle cell. Scale bar, 10 μm. **(E)** Left view of midsagittal section of an unstained 16-hpf larva acquired by quantitative X-ray tomographic microscopy. Vertical arrow (x’) indicates a high-density structure, and the position of cutting plane in **(F)**. Scale bar, 10 μm. **(F)** Anterior view of a tail vertical cross-section from an unstained 16-hpf larva. Scale bar, 10 μm. **(G)** Dorsal view of osmium-stained 16-hpf larva shown in **(B–D)**, pseudo-colored green. The midsagittal plane is manually drawn in red in the tail. Scale bar, 50 μm.

To conduct morphological analysis of bilateral symmetry of *Ciona* tadpole larvae, we first examined the midsagittal plane. Specifically, we harvested larvae at 16–18 h post fertilization (hpf) and fixed them with osmium tetroxide as is used in electron microscopy protocols. Under bright field microscopy, swimming larvae at 18-hpf showed an otolith and ocellus in the trunk and the notochord in the tail (Figure 1A, St. 26) (Hotta et al., 2007). Specimens were then imaged with quantitative X-ray tomographic microscopy, which allows imaging of biological samples (Matsuo et al., 2015; Takano et al., 2020; Kuroda et al., 2021), to visualize the three-dimensional density distribution in whole larvae at cellular resolution. As expected,16-hpf larvae showed more rounded trunks relative to 18-hpf larvae (Figure 1B, St. 25). Along the midsagittal plane, the otolith in the trunk, the anterior notochord, and a longitudinal ventral structure all exhibited high density, indicative of accumulation of calcium, zinc, or other metal elements (Figure 1C, indicated by ot, nc, and x). Vertical cross-sections confirmed accumulation of high-density material or mineralization in the longitudinal ventral structure, which was either within or around the endodermal strand (Figure 1D). To exclude the possibility that high-density material was a product of osmium tetroxide fixation, we analyzed control samples without osmium fixation. In the absence of osmium, we reproducibly observed high density of the otolith, as well as of the ventral structure (indicated by x’) in midsagittal sections (Figure 1E) and vertical cross-sections (Figure 1F), while high density of the anterior notochord, which was consistently observed in osmium-fixed larvae, was not apparent in unstained larvae. Thereafter, the otolith and the longitudinal ventral structure served as midsagittal markers, allowing detection of the midsagittal plane and dorsoventral orientation in silico (Figure 1G).

### Bilaterality of Muscle Cell Nuclei in the Larval Tail

**FIGURE 2:**
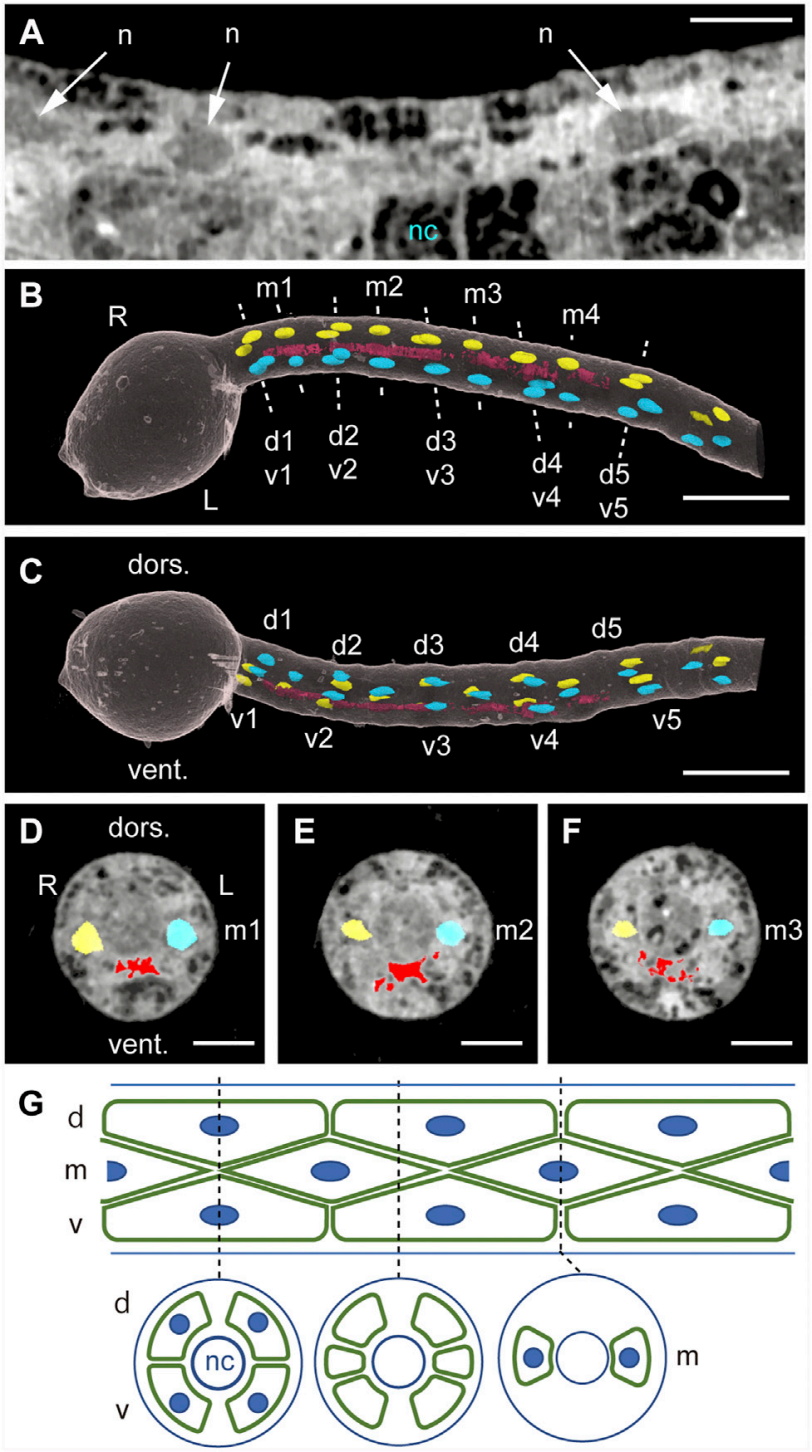
Bilateral positions of muscle cell nuclei as analyzed by quantitative X-ray tomographic microscopy. **(A)** Horizontal section of the tail in a *Ciona* 16-hpf larva. n, muscle cell nucleus. nc, notochord. Scale bar, 10 μm. **(B)** Dorsal view of positions of muscle cell nuclei pseudo-colored blue (left side) and yellow (right side). red, ventral high-density structure. Three rows of dorsal (d), middle (m), and ventral (v) muscle cells are numbered from anterior to posterior. Scale bar, 50 μm. **(C)** Left lateral view. dors., dorsal. vent., ventral. Scale bar, 50 μm. **(D–F)** Anterior views of vertical cross-sections at nuclei of m1, m2, and m3 muscle cells. Scale bar, 10 μm. **(G)** Schematic lateral and cross-sectional views of muscle cells (green) and nuclei (blue).

Elongation of the tail occurs between 9 and 12 hpf at 18°C in *Ciona* larvae without cell proliferation and is accompanied by dramatic increases in the distance between nearest-neighbor muscle cell nuclei (Passamaneck et al., 2007). We asked whether muscle cell nuclei on both sides of the midsagittal plane are bilaterally located in the fully extended tail at 16 hpf. Horizontal sectioning in silico revealed muscle cell nuclei as low-density oval areas (Figure 2A). When we pseudo-colored left-side (blue) and right-side (yellow) nuclei and inspected them from the dorsal side of the body we observed that 14 primary muscle cells were aligned in three rows—dorsal, middle, and ventral—on each side, as expected (Nishino et al., 2010) (Figure 2B). In the left lateral view, left side nuclei could be roughly superimposed on right side nuclei (Figure 2C). Moreover, a dorsal view and evaluation of cross-sections at nuclei of muscle cells m1, m2, and m3 (Figures 2D–F) confirmed gross bilateral symmetry in the position of muscle cell nuclei in the elongated tail. Figure 2G schematically shows lateral and cross-sectional views of muscle cells and nuclei in the larval tail.

### Light-Sheet Fluorescence Microscopy Reveals Left-Right Asymmetry in Myofibril Orientation

**FIGURE 3:**
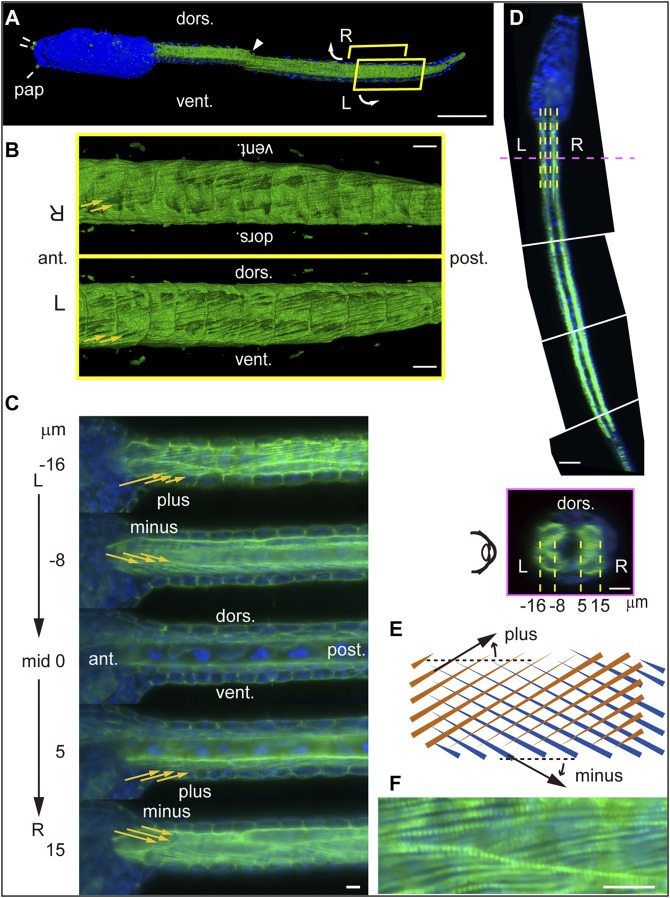
Lightsheet microscopy analysis of myofibril orientation in *Ciona* tadpole larvae. **(A)** Left side view of an entire *Ciona* larva at 18 hpf stained with phalloidin (green) and DAPI (blue). pap, adhesive papillus. dors., dorsal. vent., ventral. Arrowhead indicates visible seam between two stitched 3D images. Curved arrows indicate ventral split of the tail shown in **(B)**. Scale bar, 50 μm. **(B)** External view of phalloidin-stained tail within R and L yellow boxes in **(A)**. Yellow arrows indicate fine lines detected. Scale bar, 10 μm. **(C)** Parasagittal sections (from top to bottom: −16, −8, 0, 5 and 15 μm from the midsagittal plane). Scale bar, 10 μm. **(D)** **(top)** Dorsal view of mid-horizontal plane of *Ciona* larva composed of four different images. Dashed yellow and magenta lines indicate positions of parasagittal- and cross-sections shown in **(C)** and **(D)**, respectively. Scale bar, 50 μm. **(bottom)** Posterior view of a vertical cross-section. Dashed yellow lines indicate positions of parasagittal sections shown in **(C)**. Scale bar, 10 μm. **(E)** Schematic microscopic views in front (brown) and back (blue) planes of fine lines angled in plus or minus orientations relative to the longitudinal axis of the tail. **(F)** Higher magnification view of parasagittal section at the left lateral layer (−16 μm from the midsagittal plane). Scale bar, 10 μm.

To examine bilaterality of subcellular structures in muscle cells, we stained tadpole larvae at 18 hpf with fluorescent phalloidin, which binds to polymerized F-actin. Imaging of whole larvae using light-sheet fluorescence microscopy revealed phalloidin-positive structures in the trunk and tail, including three adhesive papillae at the anterior end, namely, two bilateral dorsal ones and a ventral structure in the midsagittal plane (Figure 3A). External views of the ventrally cut-open tail revealed angled lines, which were surprisingly left-right asymmetric (Figure 3B). We further examined parasagittal sections of specimens by viewing from the left side of the larva (Figure 3C). In the left muscle cells, we detected multiple fine lines angled relative to the longitudinal axis of the tail (Figures 3C,D, −16 and −8 μm), and line orientations were plus and minus (Figure 3E) at lateral and medial layers, respectively. Progressively, beyond the midsagittal plane and in right muscle cells, we again observed plus and minus orientations of lines at medial and lateral layers, respectively (Figures 3C,D, 5 and 15 μm). This asymmetric “plus-minus-plus-minus” pattern was unexpected and different from “plus-minus-minus-plus” or “minus-plus-plus-minus” bilaterally symmetric patterns. The angle of these lines resembles patterns seen in muscle cell myofibrils in the tail of ascidian tadpole larvae (Grave, 1920; Berrill and Sheldon, 1964; Ohtsuka and Okamura, 2007; Passamaneck et al., 2007). A higher magnification image of a region 16 μm left of the midsagittal plane (Figure 3C, top) revealed a pattern resembling that of striated muscle fibers (Figure 3F), suggesting that these lines are myofibrils.

### Chorionated Embryos Also Produce Asymmetric Myofibrils

Although we consistently observed the plus-minus-plus-minus asymmetric pattern of myofibrils, we routinely dechorionated eggs before fertilization to increase embryo visibility. Since dechorionation affects organ lateralization of the ocellus, heart, and brain (Katsumoto et al., 2013; Palmquist and Davidson, 2017; Kourakis et al., 2021), we next fertilized eggs within the chorion and allowed them to develop and hatch as tadpole larvae. When we stained fully developed tadpole larvae at 24 hpf with phalloidin and analyzed myofibril orientation, all twelve larvae examined exhibited the asymmetric “plus-minus-plus-minus” pattern (Supplementary Figure S1, Supplementary Video S1). These data indicate the existence of symmetry-breaking myofibrils on both sides of the bilaterally symmetric locomotor system of *Ciona* larvae.

### Myofibrils Exhibit Left-Right Asymmetry in Dorsal View

**FIGURE 4:**
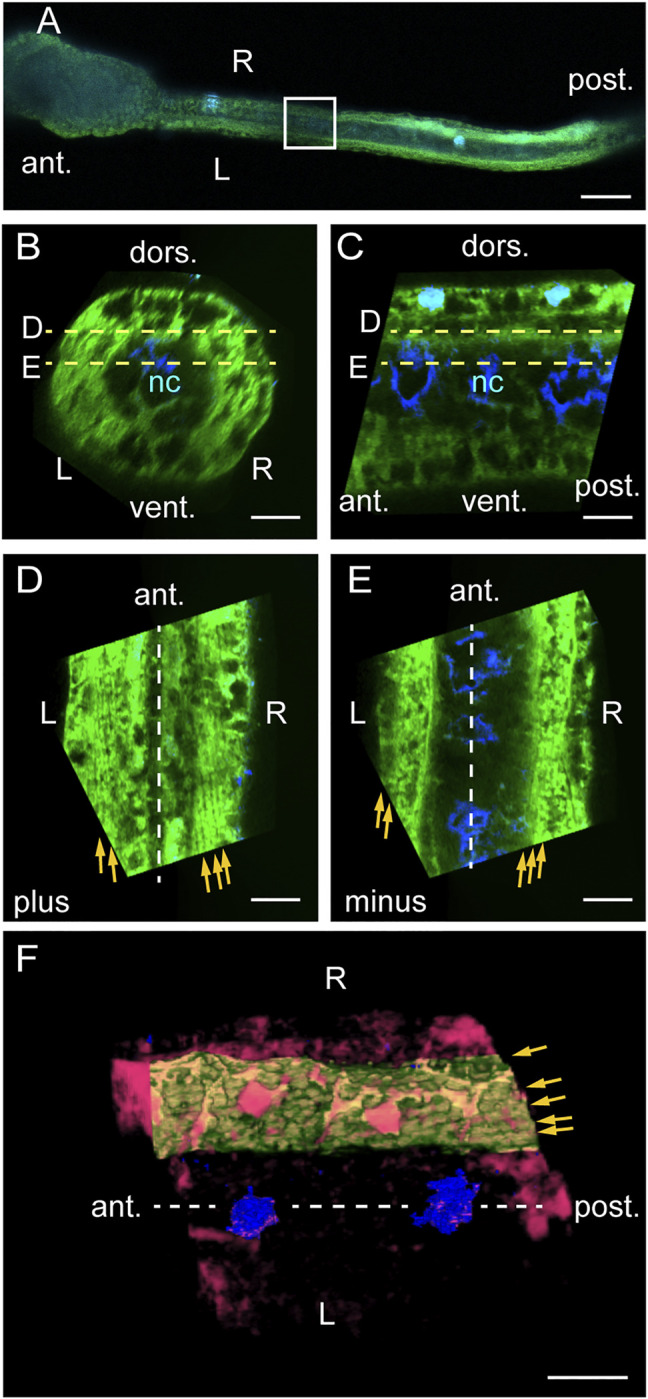
Myofibrils exhibit left-right asymmetry in dorsal view. **(A)** Dorsal view of a phalloidin/DAPI-stained *Ciona* larva at 24 hpf. Boxed photo-bleached area is analyzed in **(B–F)**. Scale bar, 50 μm. **(B)** Posterior view of a cross-section in silico. Scale bar, 10 μm. **(C)** Left view of a midsagittal section in silico. Scale bar, 10 μm. **(D)** Dorsal view of a horizontal section in silico through the top of dorsal muscle cells at level D in **(B, C)**. Yellow arrows indicate fine lines detected. Scale bar, 10 μm. **(E)** Horizontal section through the bottom of dorsal muscle cells at level E in **(B, C)**. Scale bar, 10 μm. **(F)** Dorsal view of 3D-rendered muscle cells. Red, high intensity phalloidin signals above a certain threshold. Green, a left dorsal muscle cell segmented in silico. Blue, DAPI. Scale bar, 10 μm. The midsagittal plane is indicated by dashed lines. Brightness was adjusted in the left and right halves of the image separately in **(D, E)**.

We next examined myofibrils horizontally using confocal laser scanning microscopy after phalloidin staining. A dorsal view of 24-hpf larva revealed intensely stained muscle cells and the less-intensely stained central notochord (Figure 4A). Vertical cross-sections (Figure 4B) and midsagittal sections (Figure 4C) showed two horizontal levels labelled D and E at which dorsal and ventral muscle cells were analyzed in Figures 4D,E, respectively. When viewed from the dorsal side of the larva, line orientations were plus on both left and right at level D above the notochord (Figure 4D) and minus on both left and right at level E under the notochord (Figure 4E) relative to the longitudinal axis of the tail, demonstrating left-right asymmetry. External views of 3-dimensional renderings of these images showed that each muscle cell contained myofibrils organized close to the cell surface (Figure 4F). These data suggest that myofibrils are in a multiple helix at a low helix angle (Table 1).

**TABLE 1:** Myofibril parameters.

Modality		Helix angle	Distance	Sarcomere length	Number^a^	Source
		degrees	μm	μm		
Lightsheet microscopy	2D sections	7.5 (SD 2.1)	1.32 (SD 0.06)	2.02 (SD 0.09)	n.d	Supplementary Figure S1 ^b^
						Figure 3F
Transmission electron microscopy (TEM)	2D sections	5.6 (SD 2.7)	0.96 (SD 0.21)	1.69 (SD 0.05)	25–50	Figure 5
Zernike X-ray tomographic microscopy	3D rendering	8.3 (SD 2.8)	0.88 (SD 0.22)	n.d	25–30	Figure 7

Myofibril parameters were measured from images. Mean (Standard Deviation). n.d. not determined.

a Number of myofibrils in a muscle cell counted in a cross-section perpendicular to the body axis.

b 12 larvae, 4 slices each (right and left, lateral and medial) for angle measurement.

### Transmission Electron Microscopy Reveals Myofibrils Underneath the Plasma Membrane

**FIGURE 5:**
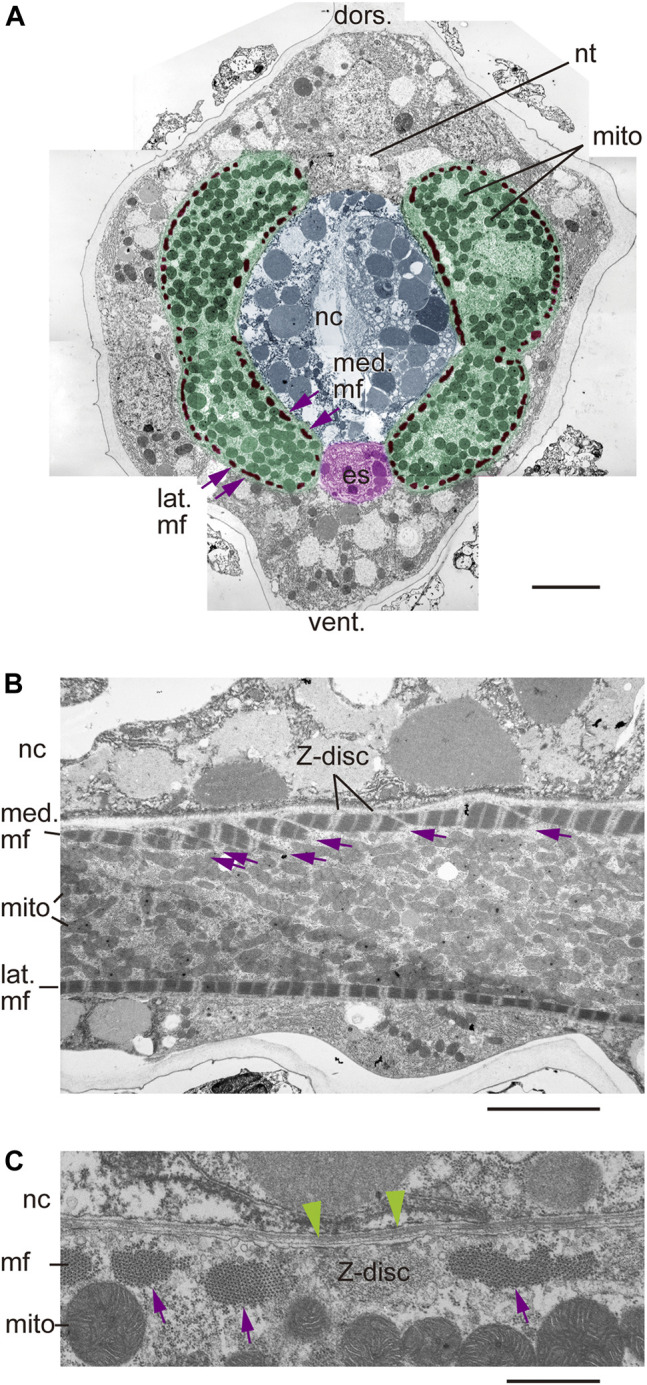
Myofibril localization underneath the plasma membrane. **(A)** Transmission electron microscopy (TEM) of a cross-section of the *Ciona* larval tail. Pseudo-colored structures are muscle cells (green), myofibrils (mf, brown), notochord (nc, blue) and endodermal strand (es, red). nt, neural tube. mito, mitochondria. dors., dorsal. vent., ventral. med., medial. lat., lateral. Nine micrographs were stitched manually. Scale bar, 5 μm. **(B)** TEM image of a semi-horizontal longitudinal section of the *Ciona* larval tail. Multiple myofibrils on the medial plasma membrane facing the notochord (med. mf) are indicated by arrows. Note clearly visible Z-discs at sarcomere borders. Scale bar, 5 μm. **(C)** Higher magnification view of a Z-disc in the cross-section shown in **(A)**. Electron dense foci (green arrowheads) that may anchor myofibrils to the plasma membrane were detected. Multiple myofibrils are indicated by arrows. Scale bar, 1 μm.

To examine myofibril location and orientation at higher resolution, we employed transmission electron microscopy. Cross-sectional analysis of the *Ciona* larval tail revealed 25–50 myofibrils arranged in a single peripheral layer underneath the plasma membrane of a muscle cell containing numerous mitochondria (Figure 5A), consistent with previous reports (Chambon et al., 2002; Meedel et al., 2007). In a semi-horizontal longitudinal section, multiple angled myofibrils were observed under the medial plasma membrane of a muscle cell when a single myofibril under the lateral plasma membrane was captured in the same section, demonstrating that medial and lateral myofibrils are not parallel but rather differentially angled (Figure 5B). Furthermore, Z-discs seen periodically between two sarcomeres within a myofibril were anchored to the plasma membrane of muscle cells in the cross-section shown in (A) (Figure 5C). These data suggest that the “plus-minus-plus-minus” asymmetric pattern of angled lines (Figures 3, 4) represents myofibrils attached at a regular interval to the inner surface of the plasma membrane.

### Zernike X-Ray Tomographic Microscopy Reveals Left-Handed Helical Myofibrils

**FIGURE 6:**
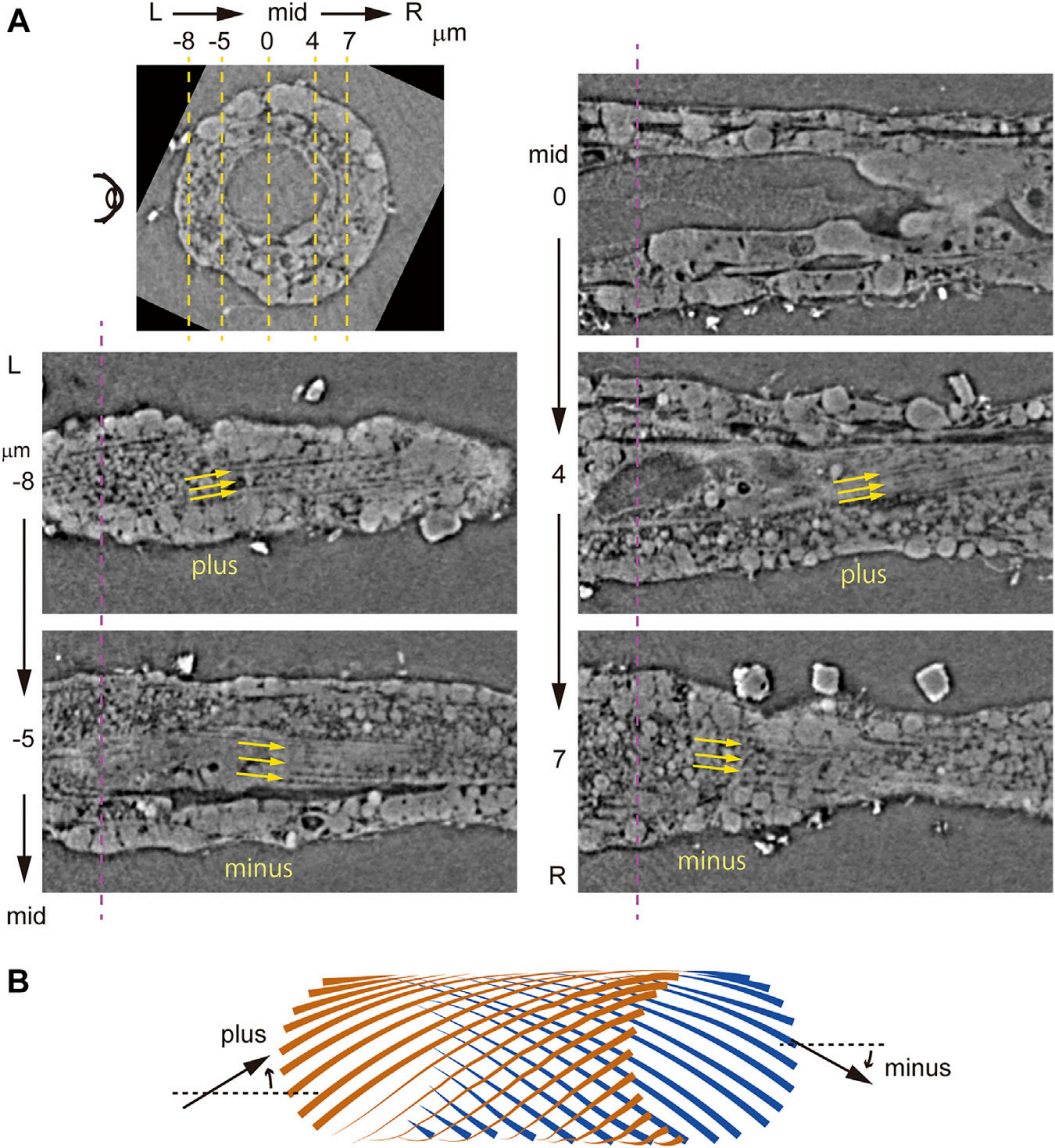
Laboratory-based Zernike X-ray tomographic microscopy of *Ciona* tadpole larvae. **(A)** Vertical cross- and parasagittal sections. Dashed magenta and yellow lines indicate positions of sections, as in Figure 3. Yellow arrows indicate myofibril orientation. **(B)** Schematic microscopic views in front (brown) and back (blue) of an inferred model of left-handed helical myofibrils. Myofibrils are angled plus or minus relative to the longitudinal axis of the tail.

To perform isotropic imaging of helical myofibrils in left and right muscle cells, we first employed laboratory-based Zernike X-ray tomographic microscopy, which emphasizes fine structures at submicron spatial resolution. To do so, we imaged a tail segment of an 18-hpf tadpole larva and detected the notochord and flanking muscle cells. As observed in fluorescence imaging, detailed inspection from the left side of the larva progressively viewing the left lateral and left medial layers to the midsagittal plane, and to the right medial and right lateral layers, revealed myofibrils inclined with the “plus-minus-plus-minus” asymmetric myofibril pattern (Figure 6A). Independently, we employed the synchrotron-based Zernike X-ray tomographic microscopy and obtained essentially identical results (Supplementary Figure S2). Although the observations of multiple planes parallel to the longitudinal axis of the tail suggested a “left-handed helix” of myofibrils in bilateral muscle cells (Figure 6B), direct visualization of helical myofibrils using fluorescence imaging had been hampered by insufficient resolution in the depth direction (*z*-axis).

**FIGURE 7:**
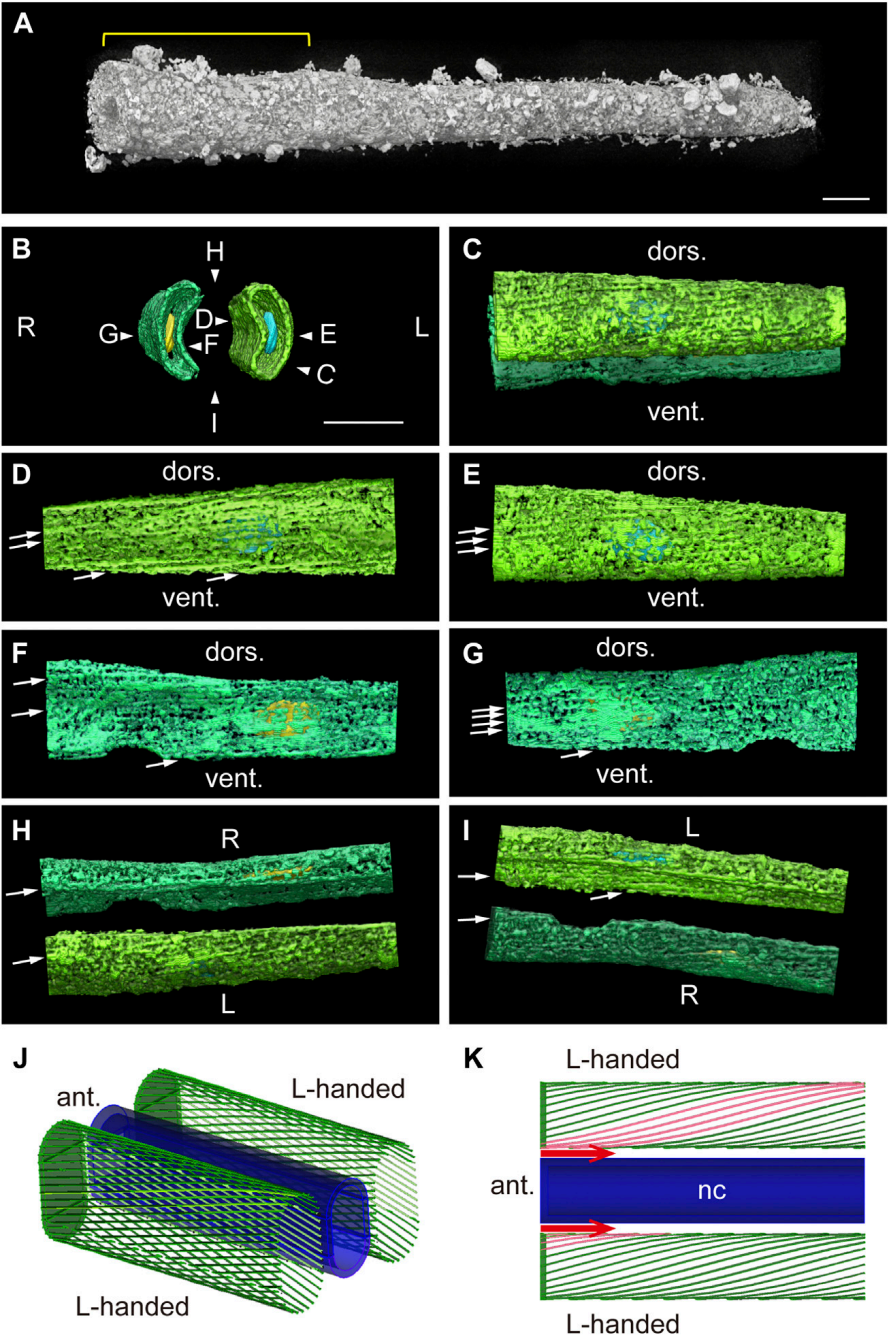
Bilaterally asymmetric left-handed helical models of myofibrils. **(A)** Laboratory-based Zernike X-ray tomographic microscopy of a *Ciona* tadpole larva near the end of the tail. The region analyzed in **(B–I)** is indicated with a yellow line. Scale bar, 10 μm. **(B)** Anterior view of the plasma membrane of muscle cells (green) and nuclei. Pseudo-colors: right nucleus, yellow; left nucleus, blue. Scale bar, 10 μm. View directions of **(C–I)** are indicated. **(C)** Left view. **(D)** Medial surface of the left muscle cell. The arrow indicates the direction of a myofibril. **(E)** Lateral surface of the left muscle cell. **(F)** Medial surface of the right muscle cell. **(G)** Lateral surface of the right muscle cell. **(H)** Dorsal view. **(I)** Ventral view. **(J)** Schematic image of two muscle cells containing left-handed (L-handed) helical myofibrils paired on either side of the notochord (dark blue). **(K)** Schematic dorsal view of myofibrils in left and right muscle cells. The model represents how neuronal stimulation (red arrows) could result in asymmetric contraction. nc, notochord. See also Supplementary Video S2.

To overcome this difficulty, we focused on an isotropic laboratory-based Zernike X-ray tomographic microscopy of the tail at 18 hpf (Figure 7A). We extracted the inner surface of the cell membrane, where myofibrils were located (Figure 5) using CT slice images with a binary mask (Figures 7B,C). Medical and lateral views of the left (Figures 7D,E) and right (Figures 7F,G) muscle cells clearly show fibrils in the plus orientation. Dorsal and ventral views show myofibrils in the plus orientation for both left and right muscle cells demonstrating left-right asymmetry (Figures 7H,I). Since external views from every angle show plus-oriented myofibrils (Supplementary Video S2), we conclude that myofibrils are in the form of left-handed helix, as shown schematically in Figure 7J. Finally, to examine whether the oblique fibers observed using fluorescence microscopy or TEM are identical to the helical structures observed using X-ray tomography, we measured the angle of myofibrils relative to the body axis, distance between parallel fibers, the sarcomere length and number of myofibrils in a muscle cell cross-section perpendicular to the body axis, and compared these parameters among the different imaging methodologies (Table 1). These parameters, which may change during contraction and are prone to fixation artifact, were roughly comparable based on all three analyses. These data support our conclusion that we are observing the identical structures, namely, helical myofibrils, using different microscopic techniques. It is worth noting that we have observed only left-handed, never right-handed, helical myofibrils after analyzing numerous *Ciona* larvae in this, and other on-going studies. These observations provide a structural basis for asymmetric left-right contraction of muscles following neural stimulation (Figure 7K).

## Discussion

The locomotor system of the *Ciona* tail is characterized by bilateral muscles seen on both left and right sides inducing bilaterally symmetric and asymmetric flexion (Nishino et al., 2011; Rudolf et al., 2019). Here, we asked whether the *Ciona* tail is bilaterally symmetric on cellular and subcellular scales and observed the presence of symmetry-breaking left-handed helices of myofibrils in bilateral muscle cells.

Using quantitative X-ray tomographic microscopy, we first identified a useful ventral midplane marker in the tail of *Ciona* larva. Specifically, a ventral longitudinal high-density structure was found within or around the endodermal strand in the tail. Since dorsoventral orientation is not always immediately evident, observation of ventral high density facilitated determination of body axes. We observed muscle cell nuclei positioned bilaterally on each side of the notochord. Paraxially-positioned muscle cells drive tail extension in association with the axially-positioned notochord (Passamaneck et al., 2007). Therefore, cell-cell interactions between the medial surface of a muscle cell and the lateral surface of notochord cells may constitute the cellular basis for mirrored positioning of muscle cells in the *Ciona* tail.

An unexpected finding reported here is the bilaterally asymmetric handedness of helical myofibrils under the plasma membrane of muscle cells. Hypothetically, a fully bilateral tail could be achieved by pairing muscle cells with left- and right-handed myofibrils (or vice versa) on each side of the notochord. However, we found that both left-right paired muscle cells contain left-handed helical myofibrils, breaking bilateral symmetry across the notochord in larvae derived from both dechorionated and chorionated eggs. Doubly left-handed configurations likely allow asymmetric contraction, which might generate rotational swimming patterns without complex input from the nervous system. Non-random myofibrillar helicity seen in larvae from dechorionated eggs suggests that helical orientation is determined independently of chorionation, neurula rotation and left-specific Nodal expression (Nishide et al., 2012; Yamada et al., 2019). How left-handed helical myofibrils are generated in *Ciona* muscle cells awaits future studies.

Some argue that “building block molecules” such as amino acids and proteins are chiral and that homochirality of these molecules leads to cellular chirality and left-right asymmetry of the animal body (Levin and Mercola, 1998; Inaki et al., 2016). Indeed, chirality of cells, namely left- or right-handedness, in multicellular organisms including vertebrates, is evident *in vitro* (Wan et al., 2011; Tee et al., 2015). *In vivo*, cells in the developing chicken myocardium are chiral, as evidenced by rightward polarization of the Golgi complex (Ray et al., 2018). It is plausible that generation of cells with single myofibrillar chirality is more cost-effective than establishment of cells with two different myofibrillar chiralities. Examination of Golgi positioning relative to the nucleus in *Ciona* muscle cells would reveal whether homochirality occurs at the whole-cell level or is limited to helical myofibrils.

Our analyses reveal that certain paired muscle cell nuclei are grossly positioned in a mirror image across the midsagittal plane, and that myofibril orientation occurs in a left-handed helix on both sides of the larval tail. This study demonstrates that the apparent bilaterality of the *Ciona* larval tail is built upon cellular homochirality, namely, upon cells with single handedness.

## Data Availability

The raw data supporting the conclusion of this article will be made available by the authors, without undue reservation.
