# Supplementary figures and images for: Bilaterally Asymmetric Helical Myofibrils in Ascidian Tadpole Larvae
Source: Front Cell Dev Biol. 2021 Dec 7;9:800455. doi: 10.3389/fcell.2021.800455 (PMC8688927; doi:10.3389/fcell.2021.800455)

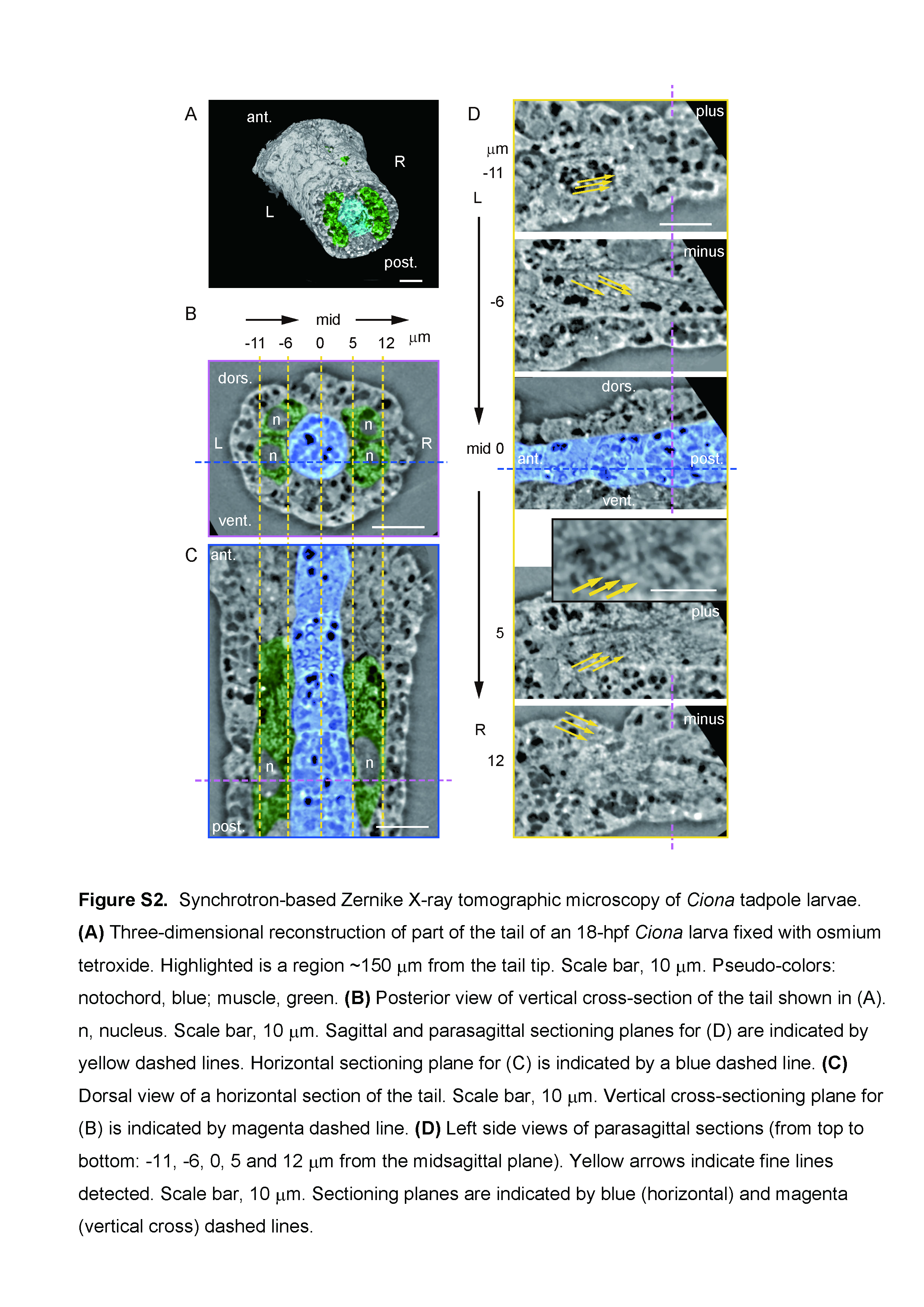

Supplement: Supplementary file 1 [file Image2.TIF]

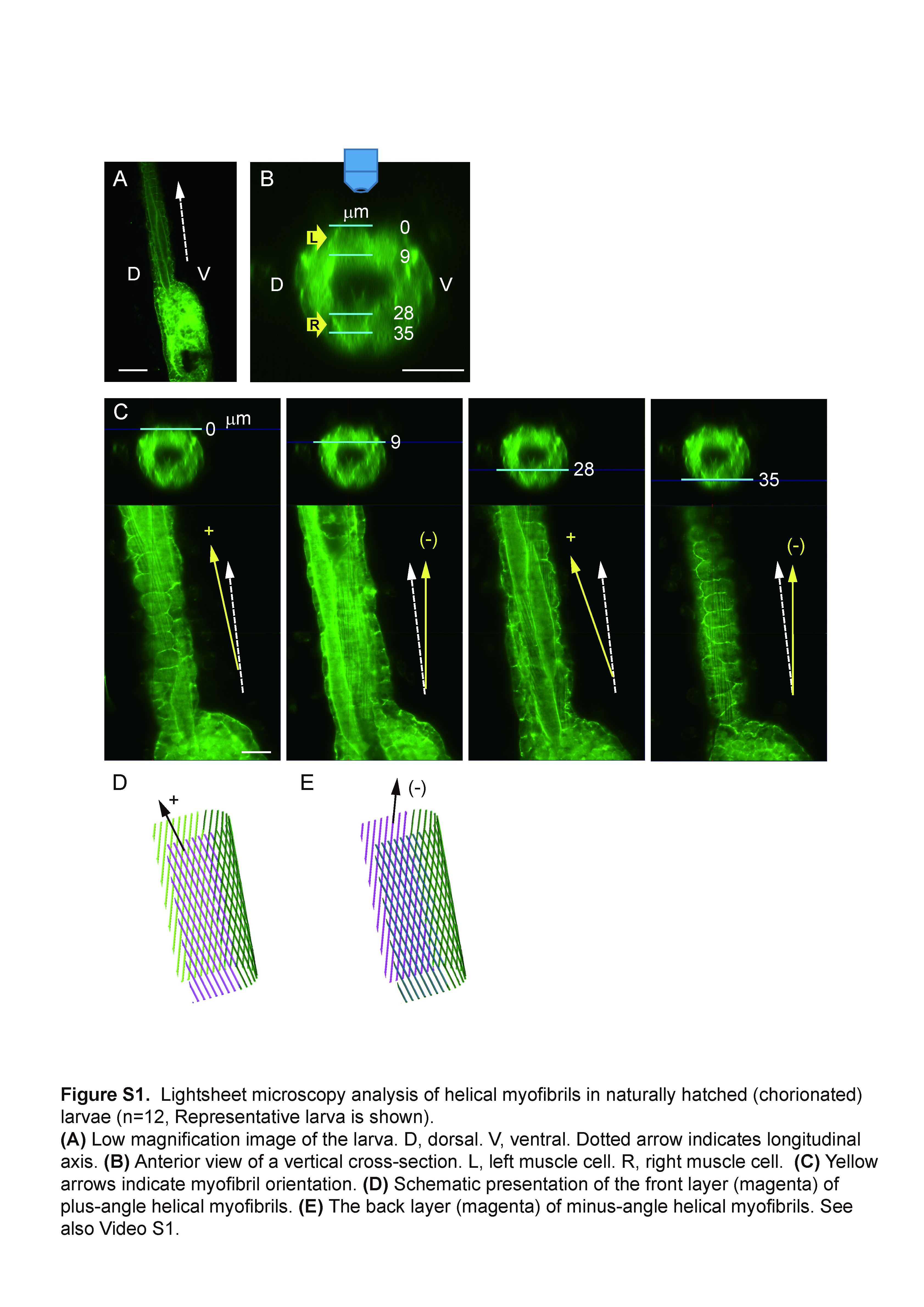

Supplement: Supplementary file 2 [file Image1.TIF]
